# Differential COVID-19 testing, admissions, and mortality for Arab Americans in Southern California

**DOI:** 10.1371/journal.pone.0267116

**Published:** 2022-04-14

**Authors:** Nadia N. Abuelezam, Kristina L. Greenwood, Sandro Galea, Raed Al-Naser

**Affiliations:** 1 Boston College William F. Connell School of Nursing, Chestnut Hill, Massachusetts, United States of America; 2 Sharp HealthCare Center for Research, San Diego, California, United States of America; 3 Boston University, School of Public Health, Boston, Massachusetts, United States of America; 4 Sharp Grossmont Hospital, La Mesa, California, United States of America; Azienda Ospedaliero Universitaria Careggi, ITALY

## Abstract

**Background:**

Understanding of COVID-19 acquisition and severity risk in minoritized groups is limited by data collection on race and ethnicity; very little is known about COVID-19 risk among Arab Americans in the United States.

**Purpose:**

To quantify whether Arab Americans in the El Cajon region of California experienced differential levels of SARS-CoV-2 infection, severity and mortality when compared to other racial/ethnic groups.

**Methods:**

A retrospective study was conducted using Sharp Grossmont Hospital’s electronic medical records. Patients were included in the study if they were: 18 years of age or older, tested for SARS-CoV-2, admitted for COVID-19 infection, or had COVID-19 listed as a cause of death between March 1, 2020 and January 31, 2021. The primary outcomes of interest were a positive COVID-19 test result, admission to the hospital due to COVID-19, and in hospital COVID-19 related mortality. Comparisons were made across racial/ethnic groups using chi-squared statistics and logistic regression models adjusted for sociodemographics, comorbidities, and time from March 2020.

**Results:**

Arab Americans had greater odds of testing positive for SARS-CoV-2 than non-Hispanic White (adjusted odds ratio, AOR: 3.83, 95% confidence interval, CI: 3.29, 4.46) and non-Hispanic Black (AOR: 2.34, 95% CI: 1.91, 2.88) patients but lower odds of admission (AOR: 0.47, 95% CI: 0.36, 0.63) and in-hospital mortality (AOR: 0.43, 95% CI: 0.28, 0.65) than Hispanic patients.

**Conclusions:**

There were distinct patterns for COVID-19 infection, severity, and mortality for Arab Americans in Southern California. Without a dedicated ethnic identifier, COVID-19 disparities facing Arab Americans will continue to go undocumented.

## Introduction

Disparities in SARS-CoV-2 infection, severity, and mortality have been documented between racial and ethnic minority groups and non-Hispanic Whites in the United States since March 2020 [1–3]. In California, Black individuals had higher odds of hospitalization [4] and mortality due to COVID than White individuals [5]. Latinx individuals in California were found to be over represented in COVID-19 case counts but underrepresented in testing and vaccine access [6]. However, our understanding of racial/ethnic differences in COVID-19 incidence, severity, and mortality in California is restricted by available data that uses a limited number of racial/ethnic signifiers. As a result very little is known about how COVID-19 has affected other minoritized groups, including for example, Arab Americans [7].

Arab Americans are individuals who have a cultural, ethnic, or linguistic origin in the Middle East and North Africa. It is estimated that there are over 3.5 million Arab Americans residing in the United States with the largest population residing in California [8]. Arab Americans are not easily identifiable from standard surveys and electronic health records due to their racial categorization as White according to the federal government [9–11]. Much of the research on Arab American health has focused on ethnic enclaves, like Dearborn Michigan, where large numbers of Arab Americans live in close geographic proximity [12]. There are a number of reasons to believe that Arab Americans may be at increased risk for SARS-CoV-2 acquisition, severity and mortality including large household sizes, occupation type, and known lack of engagement with preventive services [7].

The city of El Cajon in the east side San Diego county is a known Arab ethnic enclave. Ethnic enclaves are concentrated populations of one ethnic minority group that are often created through chain migration [13, 14]. While the majority of the original immigrants to the El Cajon community were from Iraq (mostly Chaldeans), many recent immigrants are from Syria, Egypt, and Palestine. El Cajon is one of three cities, with La Mesa and Santee, that comprise what is referred to as East County, San Diego. Between 50–60,000 residents are believed to be of Arab descent in East County. Most of these residents are recent immigrants who came to the United States as refugees or asylum seekers. One of the primary healthcare centers in the area that caters to the El Cajon community is Sharp Grossmont Hospital in La Mesa. Sharp Grossmont hospital offers a unique opportunity to assess the potential impact of COVID-19 on a dense Arab American community.

The aim of this study was to quantify whether Arab American patients in the El Cajon region of California were experiencing higher levels of SARS-CoV-2 infection, COVID-19 severity and in-hospital mortality when compared to non-Hispanic White, Black, Hispanic and Asian patients at Sharp Grossmont Hospital. This analysis represents the first quantification of disparities in COVID-19 testing, admissions, and mortality among Arab Americans nationally.

## Methods

### Study design

A retrospective study was conducted using Sharp Grossmont Hospital’s electronic medical record. Patient records were included in the study if: patients were 18 years of age or older, were tested for SARS-CoV-2, were admitted for COVID-19 infection, or had COVID-19 listed as a cause of death between March 1, 2020 and January 31, 2021. This research was approved by the Sharp HealthCare Institutional Review Board. De-identified data were analyzed and therefore consent was not obtained. It was necessary for the Sharp HealthCare Clinical Analytics team to have initial access to the last name of the patients in order to determine potential inclusion in the Arab American group. Subsequent to coding patients (using numerical values) into the respective racial/ethnic groups, the last names were deleted to produce a fully de-identified data set. The research members employed by Sharp HealthCare’s Clinical Analytics team accessed the electronic medical records during the time period of December 2020 through March 2021.

### Primary exposures and outcomes

The primary exposure of interest was race and ethnicity. Arab American ethnicity was determined in one of three ways: if place of birth was one of 22 Arab League countries, preferred language was Arabic, or if the last name matched a surname list [15] that was applied to the data extracted from the medical record. Race and ethnicity for non-Arab American patients was determined from the medical record and was categorized as non-Hispanic White (NHW), Hispanic, non-Hispanic Black (NHB), non-Hispanic Asian (NHA), American Indian, Native Hawaiian/Pacific Islander, Other, or Unknown. Due to small sample sizes American Indian, Native Hawaiian/Pacific Islander, Other, and Unknown individuals were categorized together for all analyses.

The primary outcomes of interest were a positive COVID-19 test result, admission to the hospital due to COVID-19, and in-hospital COVID-19 related mortality. COVID-19 test results were noted in the medical record along with the date of the test. Patients were considered admitted to the hospital due to COVID-19 if they had a positive test result that occurred at the same time or prior to their admission date and were noted as receiving inpatient care. In-hospital mortality was noted in the medical record.

Other data collected from the electronic medical record included sex at birth, age, visit type (inpatient, outpatient/emergency, observation), smoking status (ever smoker, never smoker), marital status (married, single/widowed/separated/divorced), insurance type (private, Medi-Cal, other public insurance, other insurance), date of SARS-CoV-2 test, date of admission, date of death, diagnosis with co-morbidities associated with the Charlson Comorbidity Index (CCI, e.g. obesity, chronic pulmonary disease, diabetes with and without complications). Zip code was extracted and compared to data from 2019 American Community Survey [16] to determine whether the individual lived in a zip code with >12% family poverty prevalence.

### Analysis

Summary statistics were calculated for all outcomes and covariates and were stratified by race and ethnicity group for all eligible individuals. Statistics were compared using chi-squared tests across all groups. Additional summary statistics and comparisons were made among those individuals who had tested positive for SARS-CoV-2 stratified by race and ethnic group.

Logistic regression models were run with testing positive for SARS-CoV-2, admission due to COVID-19, and in-hospital COVID-19 related mortality as the primary outcomes. The primary exposure variables in these analyses compared Arab American patients to NHW, Hispanic, and NHB patients. Models were adjusted for sex at birth, age group, living in a high poverty area, CCI, marital status, insurance status, months since March 2020, and obesity. All analyses were run using SAS software version 9.4 (SAS Institute Inc., Cary, NC, USA).

## Results

### Overall sample

A total of 25,216 individuals met inclusion criteria between March 1, 2020 and January 31, 2021. The largest racial/ethnic group were NHW (N = 10188, 40.4%) patients, followed by Hispanic (N = 6164, 24.4%), Arab American (N = 2744, 10.9%), NHB (N = 1902, 7.5%), and NHA (N = 641, 2.5%) patients (Table 1). Individuals who identified as American Indian, Pacific Islander, Other, or had unknown race or ethnicity accounted for 14.2% of the sample (N = 3577).

**Table 1:** Comparison of sociodemographics and outcomes across racial and ethnic groups among patients who were tested for COVID-19 in the Sharp Grossmont electronic medical record between March 1, 2020 and January 31, 2021.

	Arab American (N = 2744)	NHW (N = 10188)	Hispanic (N = 6164)	Black (N = 1902)	Asian (N = 641)	All Others (N = 3577)
**Age group, N (%)**						
*18–29*	590 (21.5)	893 (8.8)	1559 (25.3)	412 (21.7)	63 (9.8)	635 (17.8)
*30–49*	921 (33.6)	2054 (20.2)	1974 (32.0)	618 (32.5)	156 (24.3)	978 (27.3)
*50–69*	726 (26.5)	3728 (36.6)	1701 (27.6)	630 (33.1)	204 (31.8)	1129 (31.6)
*70+*	507 (18.5)	3513 (34.5)	930 (15.1)	242 (12.7)	218 (34.0)	835 (23.3)
**Sex, N (%)**						
*Male*	1266 (46.1)	4894 (48.0)	3703 (60.1)	1056 (55.5)	418 (65.2)	1700 (47.5)
*Female*	1478 (53.9)	5294 (52.0)	2461 (39.9)	846 (44.5)	223 (34.8)	1877 (52.5)
**Visit type, N (%)**						
*Inpatient*	896 (32.7)	5885 (57.8)	3258 (52.9)	957 (50.3)	407 (63.5)	1854 (51.8)
*Outpatient/Emergency*	1471 (53.6)	2283 (22.4)	2157 (35.0)	620 (32.6)	123 (19.2)	1163 (32.5)
*Observation*	377 (13.7)	2020 (19.8)	749 (12.2)	325 (17.1)	111 (17.3)	560 (15.7)
**Smoking, N (%)**						
*Ever smoker*	618 (22.8)	3856 (38.5)	1352 (22.4)	704 (37.7)	140 (22.2)	1121 (31.9)
*Never smoker*	1930 (71.1)	5236 (52.3)	4225 (69.9)	977 (52.3)	424 (67.2)	2021 (57.6)
*Missing*	165 (6.1)	920 (9.2)	466 (7.7)	188 (10.1)	67 (10.6)	370 (10.5)
**Married, N (%)**						
*Married*	1641 (61.1)	3476 (34.8)	2213 (37.3)	397 (21.6)	275 (43.9)	1163 (35.6)
*Single/Widowed/Separated/Divorced*	1047 (39.0)	6506 (65.2)	3726 (62.7)	1441 (78.4)	352 (56.1)	2108 (64.5)
**Insurance type, N (%)**						
*Private*	252 (9.2)	2109 (20.7)	1166 (18.9)	258 (13.6)	159 (24.8)	844 (23.6)
*Medi-Cal*	2056 (74.9)	2785 (27.3)	3456 (56.1)	1028 (54.1)	189 (29.5)	1394 (39.0)
*Other Public*	418 (15.2)	175 (41.6)	1407 (22.8)	579 (30.4)	285 (44.5)	1254 (35.1)
*Other Insurance*	18 (0.7)	13 (3.1)	135 (2.2)	159 (1.6)	8 (1.3)	85 (2.4)
**Living in a zipcode with >12% family poverty prevalence, N (%)**	1590 (58.6)	2591 (26.9)	2231 (37.7)	567 (32.7)	176 (27.9)	1027 (30.4)
**% of families in zipcode living in poverty, Mean (SD)**	11.3 (3.5)	8.9 (4.6)	11.3 (5.6)	10.9 (5.1)	10.4 (5.2)	9.8 (5.0)
**Obese, N (%)**	952 (38.8)	3326 (33.9)	2649 (46.1)	770 (43.4)	127 (20.6)	1271 (37.8)
**Chronic pulmonary disease, N (%)**	218 (7.9)	2095 (20.6)	712 (11.6)	429 (22.6)	93 (14.5)	530 (14.8)
**Diabetes without complications**	293 (18.0)	1512 (14.8)	1219 (19.8)	343 (18.0)	147 (22.9)	603 (16.9)
**Diabetes with complications**	154 (5.6)	765 (7.5)	507 (8.2)	170 (7.5)	73 (11.4)	272 (7.6)
**Hypertension, N (%)**	761 (27.7)	3686 (36.2)	1538 (25.0)	545 (28.7)	232 (36.2)	1066 (28.7)
**CCI—Charlson Comorbidity Index, Mean (SD)**	2.3 (2.8)	3.7 (2.9)	2.4 (2.8)	2.8 (3.0)	3.8 (3.3)	2.9 (2.9)

Differences in sociodemographic factors were observed across the race and ethnicity groups (Table 1). Most notably, the majority of Arab American patients (74.9%) were primarily insured through Medi-Cal (California’s Medicaid option). Living in a high poverty area was most common for Arab American (58.6%) followed by Hispanic (37.7%) patients. Overall, fewer Arab American patients had pre-existing health conditions than the other groups including chronic pulmonary disease, hypertension, and a lower CCI.

### COVID-19+ sample

A total of 5,513 individuals had a positive SARS-CoV-2 test between March 1, 2020 and January 31, 2021. Hispanic (N = 2041, 37.0%), NHB (N = 360, 6.5%), and Arab American (N = 1091, 19.8%) individuals were disproportionately represented in the COVID-19+ sample when compared to NHW (N = 1192, 21.6%) and NHA (N = 111, 2.0%) individuals. Arab American patients who tested positive were most commonly in the 30–49 age category (37.7%) which differed from the age distributions of positive patients in the other race and ethnic groups (Table 2). A smaller proportion of Arab American patients were admitted after a positive test (15.5%) when compared to NHW (42.0%), Hispanic (26.9%), NHB (26.4%), and NHA (46.0%) patients. A smaller proportion of Arab American patients (2.4%) experienced in-hospital mortality when compared to other ethnic groups, most notably NHA (16.2%) patients. Presenting symptoms also differed between racial and ethnic groups who tested positive (Table 2).

**Table 2:** Comparison of sociodemographics and outcomes across racial and ethnic groups among patients who tested positive for COVID-19 in the Sharp Grossmont electronic medical record between March 1, 2020 and January 31, 2021.

	Arab American (N = 1091)	NHW (N = 1192)	Hispanic (N = 2041)	Black (N = 360)	Asian (N = 111)	All Other Groups (N = 718)
**Age group, N (%)**						
*18–29*	203 (18.6)	106 (8.9)	414 (20.3)	93 (25.8)	3 (2.7)	145 (20.2)
*30–49*	411 (37.7)	236 (19.8)	662 (32.4)	113 (31.4)	24 (21.6)	220 (30.6)
*50–69*	340 (31.2)	401 (33.6)	677 (33.2)	101 (28.1)	46 (41.4)	219 (30.5)
*70+*	137 (12.6)	449 (37.7)	288 (14.1)	53 (14.7)	38 (34.2)	134 (18.7)
**Sex, N (%)**						
*Male*	532 (48.8)	573 (48.1)	886 (43.4)	159 (44.2)	42 (37.8)	355 (49.4)
*Female*	559 (51.2)	619 (51.9)	1155 (56.6)	201 (55.8)	69 (62.2)	363 (50.6)
**Admitted due to COVID, N (%)**	169 (15.5)	501 (42.0)	549 (26.9)	95 (26.4)	51 (46.0)	191 (26.6)
**Visit type, N (%)**						
*Inpatient*	217 (19.9)	595 (49.9)	703 (34.4)	115 (31.9)	56 (50.5)	246 (34.3)
*Outpatient/Emergency*	842 (77.2)	544 (45.6)	1278 (62.6)	232 (64.4)	47 (42.3)	444 (61.8)
*Observation*	32 (2.9)	53 (4.5)	60 (2.9)	13 (3.6)	8 (7.2)	28 (3.9)
**Smoking, N (%)**						
*Ever smoker*	194 (17.9)	317 (26.9)	297 (14.9)	80 (22.4)	20 (18.0)	141 (20.0)
*Never smoker*	829 (76.6)	666 (56.5)	1559 (78.2)	238 (66.7)	73 (65.8)	503 (71.3)
*Missing*	60 (5.5)	195 (16.6)	137 (6.9)	39 (10.9)	18 (16.2)	62 (8.8)
**Married, N (%)**						
*Married*	686 (64.2)	389 (33.3)	875 (44.8)	70 (19.9)	38 (35.5)	258 (33.3)
*Single/Widowed/Separated/Divorced*	383 (35.8)	779 (66.7)	1077 (55.2)	282 (80.1)	69 (64.5)	392 (60.3)
**Insurance type, N (%)**						
*Private*	99 (9.1)	272 (22.8)	463 (22.7)	61 (16.9)	30 (27.0)	199 (27.7)
*Medi-Cal*	867 (79.5)	322 (27.0)	1132 (55.5)	186 (51.7)	25 (22.5)	329 (45.8)
*Other Public*	123 (11.3)	591 (49.6)	418 (20.5)	110 (30.6)	55 (49.6)	184 (25.6)
*Other*	2 (0.2)	7 (0.6)	28 (1.4)	3 (0.8)	1 (0.9)	6 (0.8)
**In hospital mortality**	26 (2.4)	122 (10.2)	109 (5.3)	13 (3.6)	18 (16.2)	42 (5.9)
**Length of stay, days (SD)**	1.4 (4.0)	3.7 (6.3)	3.1 (7.0)	2.4 (5.9)	4.6 (8.3)	2.8 (5.9)
**Presenting symptoms, N (%)**						
*Cough*	226 (20.7)	92 (7.7)	269 (13.2)	38 (10.6)	12 (10.8)	103 (14.4)
*Diarrhea*	9 (0.8)	8 (0.7)	20 (1.0)	6 (1.7)	0 (0.0)	9 (1.3)
*Fever*	102 (9.4)	180 (15.1)	318 (15.6)	63 (17.6)	19 (17.1)	128 (17.9)
*Shortness of breath*	117 (10.7)	246 (20.7)	329 (16.2)	40 (11.2)	21 (18.9)	104 (14.5)
*Other*	637 (58.4)	664 (55.8)	1100 (54.0)	211 (58.9)	59 (53.2)	373 (52.0)
**Living in a zipcode with >12% family poverty prevalence, N (%)**	657 (60.4)	356 (30.8)	798 (40.2)	109 (31.9)	30 (37.4)	272 (39.2)
**Obese, N (%)**	375 (40.4)	382 (34.6)	885 (49.3)	158 (49.1)	15 (14.6)	271 (42.8)
**Diabetes with complications, N (%)**	27 (2.5)	96 (8.1)	126 (6.2)	28 (7.8)	12 (10.8)	34 (4.7)
**Diabetes without complications, N(%)**	200 (18.3)	207 (17.4)	488 (24.0)	72 (20.1)	32 (28.8)	135 (18.8)
**Chronic pulmonary disease, N (%)**	68 (6.2)	259 (21.8)	224 (11.0)	99 (27.7)	18 (16.2)	83 (11.6)
**Hypertension, N (%)**	292 (26.8)	419 (35.2)	542 (26.6)	91 (25.4)	52 (46.9)	199 (27.7)
**CCI—Charlson Comorbidity Index, Mean (SD)**	1.9 (2.1)	3.5 (2.8)	2.3 (2.4)	2.6 (2.8)	4.0 (3.1)	2.3 (2.5)
**Month tested positive**						
*March*	12 (1.1)	17 (1.4)	11 (0.5)	4 (1.1)	1 (0.9)	9 (1.3)
*April*	7 (0.6)	53 (4.5)	59 (2.9)	9 (2.5)	5 (4.5)	20 (2.8)
*May*	11 (1.0)	37 (3.1)	103 (5.1)	9 (2.5)	7 (6.3)	22 (3.1)
*June*	43 (3.9)	37 (3.1)	130 (6.4)	32 (8.9)	3 (2.7)	39 (5.4)
*July*	150 (13.8))	115 (9.7)	207 (10.2)	32 (8.9)	5 (4.5)	78 (10.9)
*August*	60 (5.5)	57 (4.8)	130 (6.4)	19 (5.3)	6 (5.4)	36 5.0)
*September*	23 (2.1)	49 (4.1)	96 (4.7)	17 (4.7)	3 (2.7)	20 (2.8)
*October*	62 (5.7)	49 (4.1)	90 (4.4)	13 (3.9)	1 (0.9)	22 (3.1)
*November*	229 (21.0)	140 (11.7)	233 (11.4)	46 (12.8)	17 (15.3)	80 (11.1)
*December*	324 (29.7)	326 (27.4)	514 (25.2)	84 (23.3)	32 (28.8)	176 (24.5)
*January*	170 (15.6)	312 (26.2)	467 (22.9)	94 (26.1)	31 (27.9)	216 (30.1)

### Models

In both unadjusted and adjusted models, Arab American individuals had higher odds of testing positive for COVID-19 between March 2020 and January 2021 than NHW and NHB patients at Sharp Grossmont (Table 3). Arab American patients had lower odds of being admitted after a positive COVID-19 test when compared to NHW (AOR: 0.54, 95% CI: 0.38, 0.78) and Hispanic (AOR: 0.47, 95% CI: 0.36, 0.63) patients in adjusted analyses. In adjusted analyses, Arab American patients had lower odds of experiencing an in-hospital mortality than Hispanic patients (AOR: 0.43, 95% CI: 0.28, 0.65).

**Table 3:** Logistic regression models examining differences between Arab American patients and other racial and ethnic groups on odds of testing positive for COVID-19, being admitted due to COVID-19, and in-hospital mortality for patients at Sharp Grossmont hospital between March 1, 2020 and January 31, 2021.

	Arab Americans vs. NHW	Arab Americans vs. Hispanics	Arab Americans vs. Blacks
**Odds of testing positive for COVID-19**			
*Unadjusted*	4.47 (4.01, 4.98)^#^	1.16 (1.05, 1.29) ^#^	2.81 (2.39, 3.29) ^#^
*Adjusted* *	3.83 (3.29, 4.46) ^#^	1.02 (0.90, 1.15)	2.34 (1.91, 2.88) ^#^
**Odds of being admitted due to COVID-19**			
*Unadjusted*	0.28 (0.22, 0.35) ^#^	0.51 (0.41, 0.64) ^#^	0.56 (0.39, 0.78) ^#^
*Adjusted* *	0.54 (0.38, 0.78) ^#^	0.47 (0.36, 0.63)	0.63 (0.37, 1.07)
**Odds of in hospital mortality**			
*Unadjusted*	0.60 (0.43, 0.84) ^#^	0.60 (0.42, 0.86) ^#^	1.44 (0.85, 2.41)
*Adjusted* *	0.87 (0.58, 1.31)	0.43 (0.28, 0.65) ^#^	1.19 (0.60, 2.33)

^#^ p<0.05

*Adjusted for: *sex*, *age group*, *living in a poverty area*, *CCI*, *marital status*, *insurance status*, *months since March*, *and obesity*

## Discussion

Using electronic medical records from a large hospital in Southern California, differences in COVID-19 testing, admission, and in-hospital mortality were documented for a range of ethnic groups including Arab American patients. Specifically, we find that Arab Americans had greater odds of testing positive for COVID-19 but lower odds of being admitted and dying in the hospital than other racial and ethnic groups. In the first analysis of its kind, we show that there are distinct patterns for COVID infection, severity, and mortality for this largely invisible minoritized group in Sothern California. These findings suggest that the Arab American community may have protective and resiliency factors that may support their recovery from COVID-19 that other communities and groups do not have.

Some of the most salient predictors for poor COVID-19 outcomes are age and the presence of co-morbidities [17]. Arab Americans who tested positive for COVID-19 in our sample were younger and had lower prevalence of comorbidities than NHW, Hispanic, and NHA patients. Yet, even after adjustment by age and CCI, Arab Americans still had reduced odds of being admitted with COVID-19 than NHW and Hispanic patients. This suggests that the population of Arab Americans in this sample was healthier than other racial and ethnic minority groups in the area. El Cajon is mostly comprised of recent immigrants to the United States, and it is possible that this represents a healthy migrant effect [18, 19] in this group, even if other work has not borne out the healthy migrant effect among Arab Americans [20, 21]. Because the majority of Arab Americans in this study lived in the Arab ethnic enclave of El Cajon, it is also possible that these patterns represent health of this group in an ethnic enclave. Evidence regarding the overall effects of living in ethnic enclaves on health has been mixed [22–26]. Ethnic enclaves have been found to be beneficial to first generation immigrants due to increased access to ethnic goods, social capital, and community services [27, 28] but detrimental for later generations due to low levels of resources for employment and concentrated poverty in some areas [29, 30]. While Arab Americans in our study had higher odds of testing positive for COVID-19, and had many social determinants of health that increased their risk for COVID-19 acquisition (large household size, low socioeconomic status), there seemed to be protection from increased severity (measured through admission) and in-hospital mortality for this population. This may suggest that while ethnic enclaves do not reduce risk of acquisition, there may be protection against serious disease and infection. Additionally, because more Arab Americans were married, there is the potential for supportive structures at home for Arab Americans dealing with infection, which may contribute to reduced severity. There is also the potential for bias against admission for this population whether due to differential practices by medical staff or due to resistance from patients.

It is important to contextualize our study results with that of other minoritized populations’ experiences with COVID in California and across the US. Data at the national level shows that Black Americans have higher odds of both hospitalization and mortality due to COVID-19 than White Americans [31]. While these patterns are likely to vary within various geographic contexts, they have been replicated in California within a large health system [4]. The odds of admission and mortality for Arab Americans did not differ from those for Black Americans at the same hospital suggesting the need for dedicated interventions for both minoritized groups. While differences in COVID-19 outcomes between minoritized groups and White Americans have been attributed to differences in socioeconomic status, living conditions, occupation, comorbidities, and access to healthcare [32], the impact of structural and systemic racism underlies many of these differences in opportunity and lived experiences [33].

The results of this analysis should be seen within the limitations of the methods. First, Arab Americans in this analysis were primarily identified using an Arab surname algorithm which may mean that some Arab Americans were included in the comparison groups and that others who are not Arab Americans may be categorized with this ethnic group. This surname algorithm has been used and validated in other studies [15, 34] in populations specific to California, but remains an imperfect approach to classifying Arab Americans. Second, patterns in disease incidence and severity are often influenced by acculturation and immigration variables. Due to our reliance on electronic medical records, these variables were not available for adjustment and inclusion in our analysis plan. Third, this analysis required that Arab Americans showed up to the hospital for a COVID-19 test and may not be representative of the whole Arab American population in El Cajon, specifically those who were uninsured or health illiterate. Our results can only be generalized to the population with health insurance who was comfortable attaining healthcare from this particular hospital setting. Finally, without contextualizing our results with multiple data points, biological specimens or qualitative information on patient experiences and provider practices, we cannot distinguish between the potential mechanisms explaining the disparity in admissions for this population.

In the first study of its kind, this analysis examined disparities in COVID-19 acquisition, severity, and mortality among Arab Americans as compared to other racial and ethnic minority groups in a hospital in Southern California. This work adds nuance to existing studies examining health inequities in COVID-19 incidence, severity, and mortality in California [4–6]. The study shows that dedicated interventions need to be put in place to reduce COVID-19 acquisition among Arab Americans. Future work should explore the protective factors that reduce severity and mortality in this population. Without a dedicated ethnic identifier, the health disparities facing Arab Americans will continue to go undocumented [35]. Providers and hospitals in areas with large Arab American populations would benefit from collecting data explicitly on this population to better serve the community and its needs.
